# Metal and pH-Dependent Aptamer Binding of Tetracyclines Enabling Highly Sensitive Fluorescence Sensing

**DOI:** 10.3390/bios12090717

**Published:** 2022-09-03

**Authors:** Yichen Zhao, Biwen Gao, Peihuan Sun, Jiawen Liu, Juewen Liu

**Affiliations:** Department of Chemistry, Waterloo Institute for Nanotechnology, Water Institute, University of Waterloo, Waterloo, ON N2L 3G1, Canada

**Keywords:** aptamers, biosensors, tetracycline, oxytetracycline, fluorescence

## Abstract

Tetracyclines are a widely used group of antibiotics, many of which are currently only used in veterinary medicine and animal husbandry due to their adverse side effects. For the detection of tetracyclines, we previously reported a DNA aptamer named OTC5 that binds to tetracycline, oxytetracycline, and doxycycline with similar *K*_D_’s of ~100 nM. Tetracyclines have an intrinsic fluorescence that is enhanced upon binding to OTC5, which can be used as a label-free and dye-free sensor. In this work, the effect of pH and metal ions on the sensor was studied. Mg^2+^ ions are required for the binding of OTC5 to its target with an optimal concentration of 2 mM. Other metal ions including Ca^2+^ and Zn^2+^ can also support aptamer binding. Although Mn^2+^ barely supported binding, the binding can be rescued by Mg^2+^. ITC studies confirmed that OTC5 had a *K*_D_ of 0.2 μM at a pH of 6.0 and 0.03 μM at a pH of 8.3. Lower pH (pH 6) showed better fluorescence enhancement than higher pH (pH 8.3), although a pH of 6.0 had slightly higher *K*_D_ values. Under optimized sensing conditions, sensors with limit of detections (LODs) of 0.1–0.7 nM were achieved for tetracycline, oxytetracycline, and doxycycline, which are up to 50-fold lower than previously reported. Milk samples were also tested yielding an LOD of 16 nM oxytetracycline at a pH of 6.0.

## 1. Introduction

Tetracyclines refer to a class of broad-spectrum antibiotics with a linearly fused four ring structure that inhibit bacterial protein synthesis by blocking the aminoacyl-tRNA attachment to ribosomes [1]. Chlortetracycline (CTC, market name Aureomycin) and oxytetracycline (OTC, market name Terramycin) were among the first tetracyclines approved for clinical use in the late 1940s [2]. Due to their toxicity, CTC, OTC, and tetracycline (TC) are currently used mostly in animal husbandry and veterinary medicine, although some are still used on humans [3]. Traces of these tetracyclines can be found in animal tissues or excreted into waterways to contaminate the environment [4]. Another highly popular tetracycline antibiotic is doxycycline (DOX), which has a longer half-life in the body and better absorption [5]. OTC, TC, and DOX have very similar structures.

Traditionally, the detection of tetracycline antibiotics was performed with chromatography or immunoassays. High-performance liquid chromatography (HPLC) was used to detect tetracyclines with good sensitivity but required extensive pre-treatment of samples [6]. Immunoassays, such as ELISA, have shown similar sensitivity to HPLC but are both cheaper and require less pre-treatment of samples [7]. Nanomaterials have also been developed to enhance detection [8,9,10]. In addition, the use of aptamers and aptasensors have gained popularity [11,12].

Aptamers are single-stranded oligonucleotides that are ideal for the detection of small molecules [13,14,15]. Advantages of aptamers include being amenable to in vitro selection, good selectivity, and good specificity. They can be combined with existing technologies such as fluorescence spectroscopy, electrochemistry, and nanomaterials to create new and improved sensing platforms [16]. Compared to antibodies, DNA aptamers are much more stable, cost-effective, and easier to modify. Recently, many new and high-quality DNA aptamers for various small molecules have been reported [17,18,19,20,21,22].

We recently reported a DNA aptamer named OTC5 that was isolated using the capture-SELEX method with OTC as a target molecule [23]. OTC5 has a high affinity to OTC, TC, and DOX with *K*_D_ around 100 nM. The tetracyclines are fluorescent with a large Stokes shift in water [24]. An interesting feature of the OTC5 aptamer is that it can enhance the fluorescence of the tetracyclines, which not only provides a method for detection, but also a convenient and robust way for measuring aptamer binding [25]. While other aptamers for tetracyclines have been reported before [26,27,28,29], we were the first to take advantage of the intrinsic fluorescence of tetracyclines for detection.

Tetracyclines have a number of pK_a_ values and changing pH can influence the charge of the molecule. In the previous work, we performed most of the binding assays in the selection buffer, which had a pH of 7.8. This pH is close to the pK_a2_ of the antibiotics and thus, they were a mixture of protonated and deprotonated forms. To test the effect of pH on aptamer binding and fluorescence enhancement, we herein systematically varied the pH to be either below or above the pK_a2_. At a pH of 6.0, the tetracyclines are charge neutral, whereas at a pH of 8.3, they carry one negative charge. Thus, these two pH values were studied.

In addition, tetracyclines are known to bind to divalent metal ions [30,31,32], which may also affect their fluorescence [33]. Metal ions can also affect aptamer binding [34,35]. Thus, the effect of metal ions coupled with the change of pH needs to be studied to characterize aptamer binding and related biosensors. In this work, we systematically studied the effect of pH and metal ions on the sensing of the tetracyclines by the OTC5 aptamer. At a pH of 6.0 with Mg^2+^, up to 20-fold fluorescence enhancement was achieved, allowing highly sensitive detection down to sub-nanomolar levels.

## 2. Materials and Methods

### 2.1. Chemicals

All of the DNA oligonucleotides were purchased from Integrated DNA Technologies (Coralville, IA, USA). TC, OTC, DOX, and other chemicals were from Sigma-Aldrich. Milli-Q water was used to prepare all the buffers and solutions.

### 2.2. Fluorescence Spectroscopy

The experiments were performed on a Tecan Spark microplate reader with excitation wavelength set at 370 nm and emission at 530 nm. For most titrations, 100 nM OTC, TC, or DOX was dissolved in buffer (10 mM MES, pH 6.0 or 10 mM Tris HCl, pH 8.3, 50 mM NaCl, 0 or 2 mM MgCl_2_). The OTC5 aptamer was titrated such that the final volume change was kept to be less than 10%. The solution was well-mixed after each titration and allowed to equilibrate for 1 min before reading. Similar methods were used for the measurement of OTC-dependent fluorescence with 0 or 2 µM OTC5 aptamer.

### 2.3. Isothermal Titration Calorimetry

Isothermal titration calorimetry (ITC) was performed using a MicroCal VP-ITC. An amount of 150 μM of oxytetracycline was titrated into 9 μM OTC5 aptamer. The oxytetracycline and OTC5 aptamer were diluted using the same buffer for the desired pH. At pH 6.0, 10 mM MES buffer with 2 mM Mg^2+^ was used. At pH 8.3, 10 mM Tris buffer with 2 mM Mg^2+^ was used. Background heat of titrating antibiotics into each buffer was subtracted. Data analysis was performed using the accompanying Origin software.

### 2.4. Milk Extraction and Detection

Milk (2% fat) was purchased from a local supermarket. Six 1.2-milileter milk samples were first spiked with different concentrations of OTC. To extract OTC, 30 μL HCl (1 M) were added to each milk sample, and the samples were centrifuged at 15,000 rpm (17,530× *g*) for 15 min. The supernatants were then removed and diluted 100-fold in MES (pH 6.0) buffer containing 2 mM Mg^2+^. An amount of 98 μL of this solution was then added to each well in a 96-well plate. Two blanks of buffer were also added to the plate. The base fluorescence was read using the Tecan microplate reader. Then, 2 μL of the 100 μM OTC5 aptamer (in water) were added for a final concentration of 2 μM OTC5, and the fluorescence was immediately read.

## 3. Results and Discussion

### 3.1. The Tetracyclines and the OTC5 Aptamer

**Figure 1:**
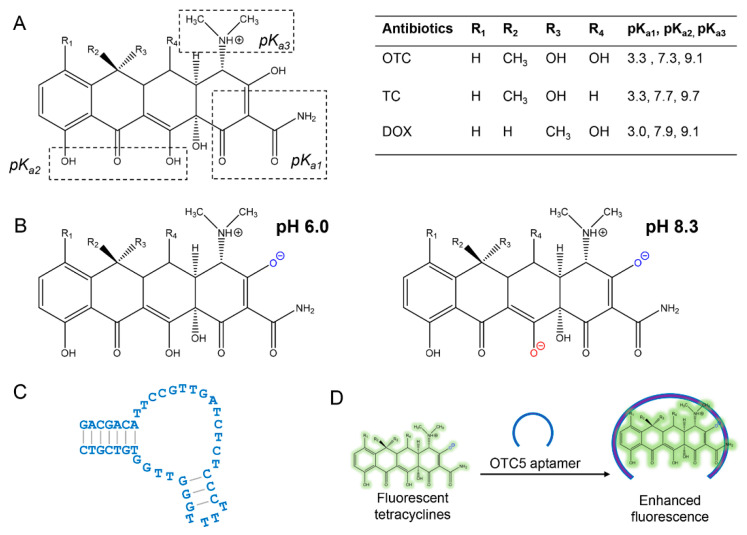
The general structure of the tetracyclines with their respective p*K*_a_ values. (**A**) p*K*_a_ values and structures adapted from ref. [16,36]. (**B**) The structure of the tetracyclines at pH 6.0 and pH 8.3. (**C**) The secondary structure of the OTC5 aptamer. (**D**) A cartoon showing the fluorescence enhancement of the tetracyclines upon aptamer binding.

The structures of OTC, TC, and DOX and their p*K*_a_ values are shown in Figure 1A. At a pH of 6.0, all three antibiotics are nearly charge neutral, whereas at a pH of 8.3, the majority of them are negatively charged (Figure 1B). The secondary structure of the OTC5 aptamer is shown in Figure 1C, which has a similar affinity for binding to these three antibiotics [23]. The majority of our previous studies was performed at a pH of 7.8, which was used for the selection of the OTC5 aptamer. Here, we studied both a pH of 6.0 and a pH of 8.3 to understand the effect of pH.

Based on the aptamer-binding-induced fluorescence enhancement of the tetracyclines, we previously developed a label-free and dye-free sensing method, as described in Figure 1D [23]. When the OTC5 aptamer was added, only the tetracyclines became fluorescent, whereas other molecules, even fluorophores, had no fluorescence change. The aptamer-binding-induced fluorescence enhancement is likely due to the more hydrophobic environment provided by the aptamer binding pocket to slow down non-radiative relaxation pathways. To achieve better sensitivity, it is desirable to have a low background fluorescence and a large fluorescence enhancement upon aptamer binding.

### 3.2. pH- and Mg^2+^-Dependent Fluorescence of the Tetracyclines

**Figure 2:**
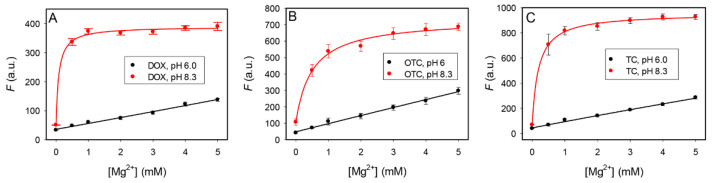
The fluorescence intensity at 530 nm of the tetracycline antibiotics at pH 6.0 and pH 8.3 in the presence of increasing concentration of Mg^2+^: (**A**) DOX, (**B**) OTC, and (**C**) TC.

We first measured the intrinsic fluorescence of the three tetracyclines as a function of Mg^2+^ concentration at two pH (Figure 2). They all had lower fluorescence at a pH of 6.0 than at a pH of 8.3. Thus, protonation of them lowered the quantum yield. At both pH levels, adding Mg^2+^ increased their fluorescence, and the fluorescence enhancement was higher at a pH of 8.3. If we fit a binding curve to these data points, the *K*_D_ for Mg^2+^ was below 0.5 mM at a pH of 8.3, whereas the *K*_D_ was higher than 5 mM at a pH of 6.0. At a pH of 6.0, the p*K*_a2_ position is protonated. It is likely that this position is involved in binding Mg^2+^ since its protonation decreased Mg^2+^ binding. These antibiotics are known to chelate divalent metal ions and such binding can enhance their fluorescence [33]. Therefore, when testing the effect of the OTC5 aptamer, we need to study the effect of pH and Mg^2+^.

For the detection of the tetracyclines using the method in Figure 1D, having a lower background fluorescence in the absence of the OTC5 aptamer is advantageous. To find the optimal Mg^2+^ concentration, we then compared the fluorescence of the samples in the absence and presence of 2 µM OTC5 aptamer, and the highest fluorescence increase occurred at above 0.5 mM Mg^2+^ (Figure S1). When the Mg^2+^ concentration was higher than 5 mM, the fluorescence enhancement also decreased. Thus, we picked 2 mM Mg^2+^ for our subsequent studies.

### 3.3. pH- and Mg^2+^-Dependent Aptamer Binding

**Figure 3:**
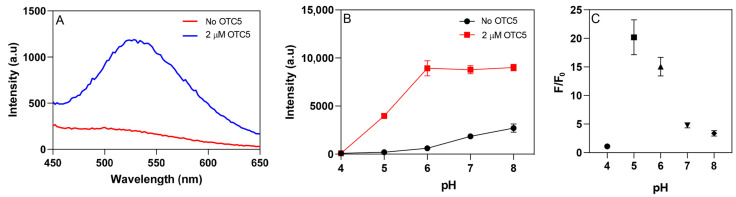
(**A**) Fluorescence spectra of 100 nM OTC in the absence and presence of 2 µM OTC5 aptamer at pH 6.0. (**B**) pH-dependent fluorescence of 1 µM OTC in the absence and presence of 2 µM OTC5 aptamer. pH 4 and 5: acetate buffer; pH 6 and 7: phosphate buffer; pH 8: Tris buffer. All with 10 mM Mg^2+^. (**C**) The fold of fluorescence enhancement at different pH levels due to the addition of the OTC5 aptamer.

We then tested the fluorescence change of OTC with the addition of the OTC5 aptamer. At a pH of 6.0, the background fluorescence of 100 nM OTC was quite low, but with the addition of 2 µM OTC5 aptamer, a large enhancement was achieved (Figure 3A). We then systematically tested the response over a broad pH range from 4 to 8 (Figure 3B). Although a pH of 5 showed the highest fold of fluorescence enhancement, a pH of 6 gave higher fluorescence intensities. In addition, TC and OTC are less stable at acidic pH [37]. Therefore, we chose a pH of 6 as our acidic pH for subsequent studies.

**Figure 4:**
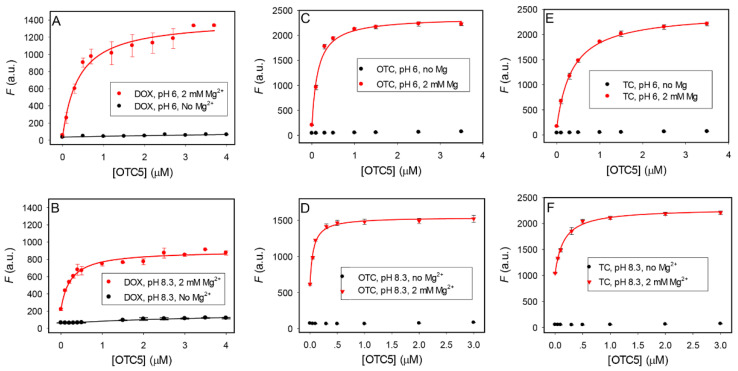
Aptamer binding curves for (**A**,**B**) DOX, (**C**,**D**) OTC, and (**E**,**F**) TC in (**A**,**C**,**E**) pH 6.0 buffer and (**B**,**D**,**F**) pH 8.3 buffers in the absence or presence of 2 mM Mg^2+^.

We then measured the OTC5 aptamer binding to 100 nM DOX at a pH of 6.0. By gradually titrating the aptamer, over 20-fold fluorescence enhancement was achieved with 3.7 µM aptamer added (Figure 4A, red line), which was much higher than that observed previously at a pH of 7.8 (only around 2-fold). The *K*_D_ was fitted to be 0.39 µM. Without Mg^2+^, the fluorescence barely changed, indicating a lack of binding. Therefore, this aptamer requires Mg^2+^ for target binding.

We then repeated this experiment at a pH of 8.3 (Figure 4B). In this case, for the sample with 2 mM Mg^2+^ added, the background fluorescence was higher and the final fluorescence was lower. The fluorescence enhancement was only 4-fold, although the *K*_D_ was slightly smaller (0.24 µM). Still, when no Mg^2+^ was added, the binding was lost and the fluorescence barely changed upon titration of the OTC5 aptamer. The same experiments were also performed for OTC (Figure 4C,D) and TC (Figure 4E,F), and the conclusions were the same. For OTC, at a pH of 6.3, the *K*_D_ for the aptamer was 0.14 µM, slightly higher than that at a pH of 8.3 (0.06 µM), but the fluorescence enhancement upon aptamer binding was much higher. For TC, at a pH of 6.3, the *K*_D_ for the aptamer was 0.36 µM, about 1-fold higher than that at a pH of 8.3 (0.16 µM).

Therefore, to use the aptamer-binding-induced fluorescence for the detection of these antibiotics, a pH of 6.0 appeared to be a better condition due to its much higher fluorescence enhancement. In all of the cases, Mg^2+^ was required for aptamer binding.

### 3.4. Aptamer Binding Assay Using ITC

**Figure 5:**
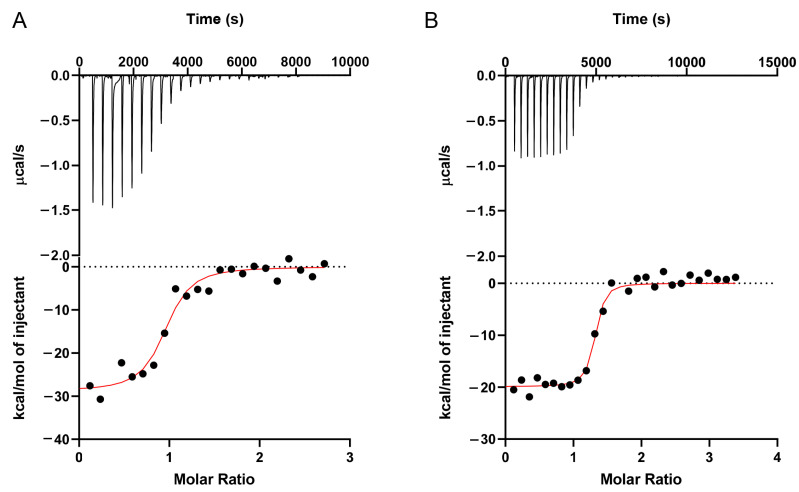
ITC traces of 150 μM OTC titrated into 9 μM OTC5 at (**A**) pH 6.0 and (**B**) pH 8.3. Both samples contained 2 mM Mg^2+^.

To further characterize the binding of the aptamers at different pH, isothermal titration calorimetry (ITC) was performed. OTC was titrated into the OTC5 aptamer at a pH of 6.0 (Figure 5A) and a pH of 8.3 (Figure 5B). The downward spikes indicated an exothermic reaction. By integrating the heat, the *K*_D_ at a pH of 6.0 was fitted to be 0.20 μM, whereas the *K*_D_ at a pH of 8.3 was nearly seven times lower at 0.03 μM (Table 1). By comparing the *K*_D_’s at a pH of 6.0 and 8.3 to the previously reported *K*_D_ at a pH of 7.8 (0.15 μM) [23], it confirmed stronger binding affinities at higher pH levels.

### 3.5. Effect of other Metal Ions

**Figure 6:**
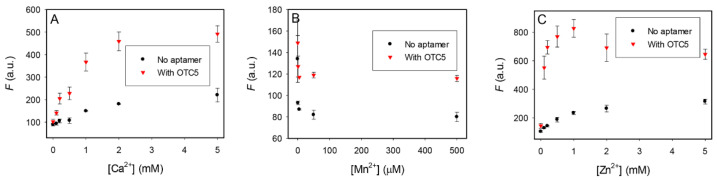
The fluorescence of 100 nM OTC alone and with 2 µM OTC5 aptamer as a function of (**A**) Ca^2+^, (**B**) Mn^2+^, and (**C**) Zn^2+^ concentration. All the experiments were run in pH 6.0 MES buffer.

After determining the importance of Mg^2+^ for aptamer binding, we then studied the effect of some other divalent metal ions. Since the behavior of these antibiotics were similar, we focused on OTC. We first replaced Mg^2+^ with Ca^2+^ (Figure 6A). Ca^2+^ can also enhance the fluorescence of OTC, indicating binding. The addition of OTC5 aptamer also enhanced the fluorescence enhancement, although the change was smaller compared to the samples with Mg^2+^. When we tested Mn^2+^, the fluorescence of OTC dropped with an increasing concentration of Mn^2+^ (Figure 6B). Although fluorescence was enhanced with the addition of the OTC5 aptamer, the increase was quite modest (less than 1-fold). With Zn^2+^, the fluorescence enhancement was higher and adding OTC5 induced a significant increase (Figure 6C). Overall, the sensor would work the best in the presence of Mg^2+^.

This could be related to the selection condition, where 10 mM Mg^2+^ was used [23]. Since the tetracyclines can bind to various metal ions [6,27,38], we reason that Mg^2+^ must be involved in bridging OTC and the aptamer instead of acting as a general salt to screen charge repulsion. Zn^2+^ and Ca^2+^ can partially replace the role of Mg^2+^ and support aptamer binding. Since the least fluorescence change by adding the OTC5 aptamer was seen with Mn^2+^, we then tested whether we can rescue the sensor performance by adding extra Mg^2+^. We performed the aptamer titration with 0.5 mM Mn^2+^ alone and with an additional 2 mM Mg^2+^ (Figure S2). With the extra Mg^2+^, 5-fold fluorescence enhancement was achieved and the overall fluorescence was also much higher. Therefore, adding extra Mg^2+^ can be a useful method for samples with unknown metal compositions.

### 3.6. Label-Free and Dye-Free Sensing of Tetracyclines

**Figure 7:**
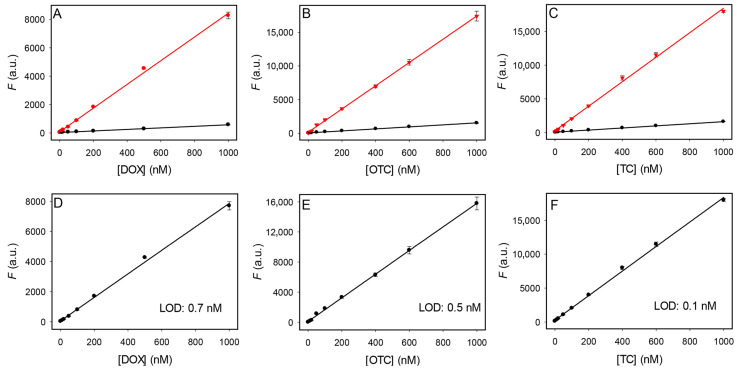
Fluorescence intensity of (**A**) DOX, (**B**) OTC, and (**C**) TC in the absence (black dots) and presence (red dots) of 2 µM OTC5 aptamer. Fluorescence difference of (**D**) DOX, (**E**) OTC, and (**F**) TC in the presence and absence of 2 µM OTC5 aptamer. The measurements were performed in pH 6 buffer with 2 mM Mg^2+^.

The above studies have also optimized the conditions to achieve better sensitivity. We then tested the sensitivity of the sensors using the method described in Figure 1D. For each analyte concentration, two measurements were performed: before and after adding 2 µM OTC5 aptamer. We chose to use 2 µM aptamer since saturated binding can be achieved at this condition, as shown in Figure 4. For all three antibiotics, we observed linear responses up to 1 µM (Figure 7A–C), and the slopes of the samples with the OTC5 aptamer were much higher. We then calculated the fluorescence difference of the two lines and plotted them in Figure 7D–F, which are the calibration curves for the tetracyclines. Based on the curves, we calculated the limit of detection (LOD) to be 0.7 nM, 0.5 nM, and 0.1 nM for DOX, OTC, and TC, respectively (3σ/slope where σ is the background variation). For comparison, in our previous condition, the LOD for OTC was 25 nM [23], and our new condition allowed a 50-fold improvement. Note that this detection method does not involve any covalent fluorophore labels or DNA staining dyes, and we solely relied on the intrinsic fluorescence of the tetracyclines.

**Figure 8:**
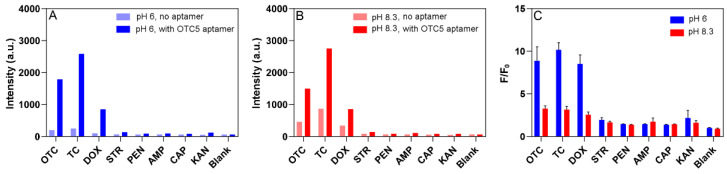
Selectivity of the OTC5 aptamer in (**A**) pH 6.0 MES buffer and (**B**) pH 8.3 Tris buffer, each with 2 mM Mg^2+^. An amount of 2 µM of OTC5 was added to 100 nM of antibiotic. Samples were scanned at an excitation wavelength of 370 nm and emission of 530 nm. (**C**) The fluorescence difference at pH 6.0 and 8.3 with and without OTC5. STR: streptomycin; PEN: penicillin; AMP: ampicillin; KAN: kanamycin.

We then tested the selectivity of the sensors against a series of different antibiotics (streptomycin, penicillin, ampicillin, chloramphenicol, and kanamycin). These antibiotics are not fluorescent at either a pH of 6.0 or 8.3, and their signals are close to zero (Figure 8A,B). OTC, DOX, and TC did show significant fluorescence increases. From highest to lowest, the fluorescence enhancement ranked TC > OTC > DOX and pH 6.0 > pH 8.3 (Figure 8C).

### 3.7. Detection in Milk

**Figure 9:**
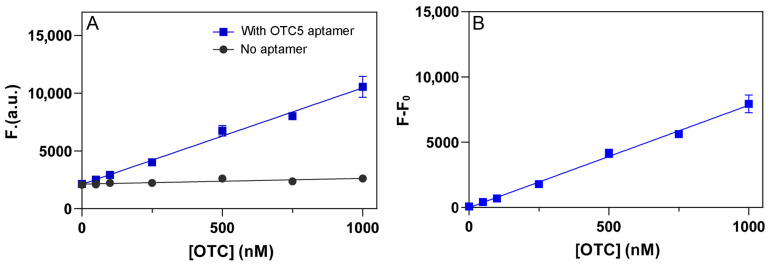
(**A**) Fluorescence intensity as a function of OTC concentration in spiked milk samples after extraction without and with 2 μM OTC5 aptamer at pH 6.0 with 2 mM Mg^2+^. (**B**) Fluorescence difference of the two curves in (**A**) as a function of OTC concentration at pH 6.0.

Finally, we tested this sensor using milk samples. Milk (2% fat) was spiked with various concentrations of OTC. Since tetracyclines are known to bind to proteins in milk, samples were first treated with HCl, centrifuged, then diluted in buffer before detection. In the treated milk, the sensor showed slightly better performance at a pH of 6.0 (Figure 9A). The LOD was calculated to be 16 nM at a pH of 6.0 (Figure 9B). This value is ~4 times lower than the LOD of 66 nM we previously reported at a pH of 7.8. We believe some of the OTC was degraded during the acid treatment [37], which may contribute to the much higher LOD value here compared to that in the buffer.

## 4. Conclusions

In this work, we investigated the pH and metal ion dependency of the OTC5 aptamer and its relevance in the fluorescent detection of three tetracycline antibiotics. The sensing performance of the OTC5 aptamer was studied at a pH of 6.0 and a pH of 8.3, at which the antibiotics were in charge neutral and negative charged states, respectively. In addition, the effect of a few common divalent metal ions was studied. When compared to other metal ions (Mn^2+^, Zn^2+^, and Ca^2+^), Mg^2+^ showed the best fluorescence increase and is required for OTC5 to bind. The optimal concentration of Mg^2+^ was around 2 mM. At a pH of 6.0, fluorescence enhancement upon binding OTC5 was shown to be greater than at a pH of 8.3 but had a higher *K*_D_. ITC experiments confirmed this by obtaining fitted *K*_D_ values of 0.2 μM and 0.03 μM at a pH of 6.0 and a pH of 8.3, respectively. Optimized sensors for TC, OTC, and DOX were tested at a pH of 6.0 with 2 mM Mg^2+^. LODs of 0.1, 0.5, and 0.7 nM were achieved, which are ~50-fold lower than previously reported. This sensor detects TC, OTC, and DOX as a group and cannot tell their difference. Other aptamers with differential binding affinities to the antibiotics are needed to detect them individually. Milk samples were tested at a pH of 6.0, and the extracted milk samples showed LODs of 16 nM. Under our optimized conditions, over 10-fold fluorescence enhancement can be readily achieved, allowing this system to be coupled with various materials for developing interesting future biosensing applications [39,40].

## Figures and Tables

**Table 1:** Thermodynamic values of the aptamers based on ITC *^a^*.

Aptamer	Target	pH	*N*	*K*_D_ (μM)	Δ*H* (cal/mol) (×10^4^)	Δ*S* (cal/mol/K)
OTC5	OTC	6.0	0.92	0.2	−2.89	−66.3
OTC5	OTC	8.3	1.27	0.03	−1.99	−32.6

*^a^* Binding ratio (*N*), dissociation constant (*K*_D_), enthalpy change (Δ*H*), and entropy change (Δ*S*) of the binding reactions are supplied.

## Data Availability

Not applicable.

## Supplementary Materials

The following supporting information can be downloaded at: https://www.mdpi.com/article/10.3390/bios12090717/s1, Figure S1: the effect of OTC5 aptamer as a function of Mg^2+^ concentration. Figure S2: rescue the sensor performance with Mn^2+^ by adding extra Mg^2+^.

## References

[1] Chopra I., Roberts M. (2001). Tetracycline Antibiotics: Mode of Action, Applications, Molecular Biology, and Epidemiology of Bacterial Resistance. Microbiol. Mol. Biol. Rev..

[2] Grossman T.H. (2016). Tetracycline Antibiotics and Resistance. Cold Spring Harb. Perspect. Med..

[3] Daghrir R., Drogui P. (2013). Tetracycline Antibiotics in the Environment: A Review. Environ. Chem. Lett..

[4] Xu L., Zhang H., Xiong P., Zhu Q., Liao C., Jiang G. (2021). Occurrence, Fate, and Risk Assessment of Typical Tetracycline Antibiotics in the Aquatic Environment: A Review. Sci. Total Environ..

[5] Smith K., Leyden J.J. (2005). Safety of Doxycycline and Minocycline: A Systematic Review. Clin. Ther..

[6] Scaria J., Anupama K.V., Nidheesh P.V. (2021). Tetracyclines in the Environment: An Overview on the Occurrence, Fate, Toxicity, Detection, Removal Methods, and Sludge Management. Sci. Total Environ..

[7] Himmelsbach M., Buchberger W. (2005). Residue Analysis of Oxytetracycline in Water and Sediment Samples by High-Performance Liquid Chromatography and Immunochemical Techniques. Microchim. Acta.

[8] Tao X., Peng Y., Liu J. (2020). Nanomaterial-Based Fluorescent Biosensors for Veterinary Drug Detection in Foods. J. Food Drug Anal..

[9] Gao W., Li P., Qin S., Huang Z., Cao Y., Liu X. (2019). A Highly Sensitive Tetracycline Sensor Based on a Combination of Magnetic Molecularly Imprinted Polymer Nanoparticles and Surface Plasmon Resonance Detection. Microchim. Acta.

[10] Wang C.-Y., Wang C.-C., Zhang X.-W., Ren X.-Y., Yu B., Wang P., Zhao Z.-X., Fu H. (2022). A New Eu-Mof for Ratiometrically Fluorescent Detection toward Quinolone Antibiotics and Selective Detection toward Tetracycline Antibiotics. Chin. Chem. Lett..

[11] Mehlhorn A., Rahimi P., Joseph Y. (2018). Aptamer-Based Biosensors for Antibiotic Detection: A Review. Biosensors.

[12] Alawad A., Istamboulié G., Calas-Blanchard C., Noguer T. (2019). A Reagentless Aptasensor Based on Intrinsic Aptamer Redox Activity for the Detection of Tetracycline in Water. Sens. Actuators B: Chem..

[13] Yu H., Alkhamis O., Canoura J., Liu Y., Xiao Y. (2021). Advances and Challenges in Small-Molecule DNA Aptamer Isolation, Characterization, and Sensor Development. Angew. Chem. Int. Ed. Engl..

[14] Alkhamis O., Canoura J., Yu H., Liu Y., Xiao Y. (2019). Innovative Engineering and Sensing Strategies for Aptamer-Based Small-Molecule Detection. TrAC Trends Anal. Chem..

[15] Ruscito A., DeRosa M.C. (2016). Small-Molecule Binding Aptamers: Selection Strategies, Characterization, and Applications. Front. Chem..

[16] Wang S., Yan X., Yang Y., Qi X., Zhao Y., Li L., Ma R., Wang L., Dong Y., Sun J. (2021). Advances and Perspectives of Aptasensors for the Detection of Tetracyclines: A Class of Model Compounds of Food Analysis. Food Chem..

[17] Nakatsuka N., Yang K.-A., Abendroth J.M., Cheung K.M., Xu X., Yang H., Zhao C., Zhu B., Rim Y.S., Yang Y. (2018). Aptamer–Field-Effect Transistors Overcome Debye Length Limitations for Small-Molecule Sensing. Science.

[18] Yang K.-A., Chun H., Zhang Y., Pecic S., Nakatsuka N., Andrews A.M., Worgall T.S., Stojanovic M.N. (2017). High-Affinity Nucleic-Acid-Based Receptors for Steroids. ACS Chem. Biol..

[19] Yu H., Luo Y., Alkhamis O., Canoura J., Yu B., Xiao Y. (2021). Isolation of Natural DNA Aptamers for Challenging Small-Molecule Targets, Cannabinoids. Anal. Chem..

[20] Huang P.-J.J., Liu J. (2022). Selection of Aptamers for Sensing Caffeine and Discrimination of Its Three Single Demethylated Analog. Anal. Chem..

[21] Luo Y., Jin Z., Wang J., Ding P., Pei R. (2021). The Isolation of a DNA Aptamer to Develop a Fluorescent Aptasensor for the Thiamethoxam Pesticide. Analyst.

[22] Tian H., Duan N., Wu S., Wang Z. (2019). Selection and Application of Ssdna Aptamers against Spermine Based on Capture-Selex. Anal. Chim. Acta.

[23] Zhao Y., Ong S., Chen Y., Jimmy Huang P.-J., Liu J. (2022). Label-Free and Dye-Free Fluorescent Sensing of Tetracyclines Using a Capture-Selected DNA Aptamer. Anal. Chem..

[24] Carlotti B., Fuoco D., Elisei F. (2010). Fast and Ultrafast Spectroscopic Investigation of Tetracycline Derivatives in Organic and Aqueous Media. PCCP.

[25] Müller M., Weigand J.E., Weichenrieder O., Suess B. (2006). Thermodynamic Characterization of an Engineered Tetracycline-Binding Riboswitch. Nucleic Acids Res..

[26] Niazi J.H., Lee S.J., Kim Y.S., Gu M.B. (2008). Ssdna Aptamers That Selectively Bind Oxytetracycline. Bioorg. Med. Chem..

[27] Niazi J.H., Lee S.J., Gu M.B. (2008). Single-Stranded DNA Aptamers Specific for Antibiotics Tetracyclines. Bioorg. Med. Chem..

[28] Berens C., Thain A., Schroeder R. (2001). A Tetracycline-Binding RNA Aptamer. Bioorg. Med. Chem..

[29] Tickner Z.J., Zhong G., Sheptack K.R., Farzan M. (2020). Selection of High-Affinity RNA Aptamers That Distinguish between Doxycycline and Tetracycline. Biochemistry.

[30] Jezowska-Bojczuk M., Lambs L., Kozlowski H., Berthon G. (1993). Metal Ion-Tetracycline Interactions in Biological Fluids. 10. Structural Investigations on Copper(Ii) Complexes of Tetracycline, Oxytetracycline, Chlortetracycline, 4-(Dedimethylamino)Tetracycline, and 6-Desoxy-6-Demethyltetracycline and Discussion of Their Binding Modes. Inorg. Chem..

[31] Lambs L., Decock-Le Reverend B., Kozlowski H., Berthon G. (1988). Metal Ion-Tetracycline Interactions in Biological Fluids. 9. Circular Dichroism Spectra of Calcium and Magnesium Complexes with Tetracycline, Oxytetracycline, Doxycycline, and Chlortetracycline and Discussion of Their Binding Modes. Inorg. Chem..

[32] Lambs L., Brion M., Berthon G. (1984). Metal Ion-Tetracycline Interactions in Biological Fluids. Part 3. Formation of Mixed-Metal Ternary Complexes of Tetracycline, Oxytetracycline, Doxycycline and Minocycline with Calcium and Magnesium, and Their Involvement in the Bioavailability of These Antibiotics in Blood Plasma. Agents Actions.

[33] Schneider S., Schmitt M.O., Brehm G., Reiher M., Matousek P., Towrie M. (2003). Fluorescence Kinetics of Aqueous Solutions of Tetracycline and Its Complexes with Mg^2+^ and Ca^2+^. Photochem. Photobiol. Sci..

[34] Lorsch J.R., Szostak J.W. (1994). In Vitro Selection of RNA Aptamers Specific for Cyanocobalamin. Biochemistry.

[35] Liu Y., Liu J. (2022). Selection of DNA Aptamers for Sensing Uric Acid in Simulated Tears. Anal. Sens..

[36] Li Y., Wang H., Liu X., Zhao G., Sun Y. (2016). Dissipation Kinetics of Oxytetracycline, Tetracycline, and Chlortetracycline Residues in Soil. Environ. Sci. Pollut. Res..

[37] Xuan R., Arisi L., Wang Q., Yates S.R., Biswas K.C. (2009). Hydrolysis and Photolysis of Oxytetracycline in Aqueous Solution. J. Environ. Sci. Health B.

[38] Guerra W., Silva-Caldeira P.P., Terenzi H., Pereira-Maia E.C. (2016). Impact of Metal Coordination on the Antibiotic and Non-Antibiotic Activities of Tetracycline-Based Drugs. Coord. Chem. Rev..

[39] Li Y., Gao H., Qi Z., Huang Z., Ma L., Liu J. (2021). Freezing-Assisted Conjugation of Unmodified Diblock DNA to Hydrogel Nanoparticles and Monoliths for DNA and Hg^2+^ Sensing. Angew. Chem. Int. Ed..

[40] Ayodele O.O., Adesina A.O., Pourianejad S., Averitt J., Ignatova T. (2021). Recent Advances in Nanomaterial-Based Aptasensors in Medical Diagnosis and Therapy. Nanomaterials.

