# Supplementary material for: Metal and pH-Dependent Aptamer Binding of Tetracyclines Enabling Highly Sensitive Fluorescence Sensing
Source: Biosensors (Basel). 2022 Sep 3;12(9):717. doi: 10.3390/bios12090717 (PMC9496453; doi:10.3390/bios12090717)
Supplement: Supplementary file 1 [file biosensors-12-00717-s001.zip › biosensors-1866605-supplementary.pdf]

Supporting Information

Metal and pH-Dependent Aptamer Binding of Tetracyclines Enabling Highly Sensitive Fluorescence Sensing

Yichen Zhao, Biwen Gao, Peihuan Sun, Jiawen Liu, and Juewen Liu *

Department of Chemistry, Waterloo Institute for Nanotechnology, Water Institute, University of Waterloo, Waterloo, ON, N2L 3G1, Canada
* Correspondence: liujw@uwaterloo.ca

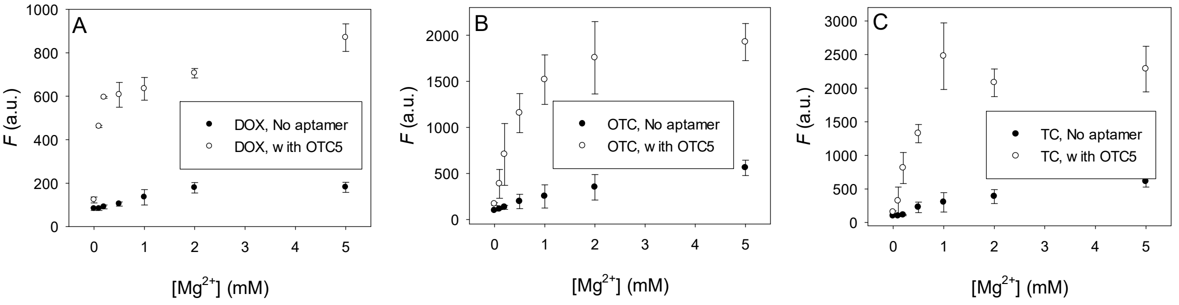

Figure S1. The fluorescence intensity of 100 nM (A) DOX, (B) OTC, and (C) TC alone and with 2 μM of the OTC5 aptamer with increasing concentration of Mg2+ in 10 mM MES, pH 6.0 with 50 mM NaCl.

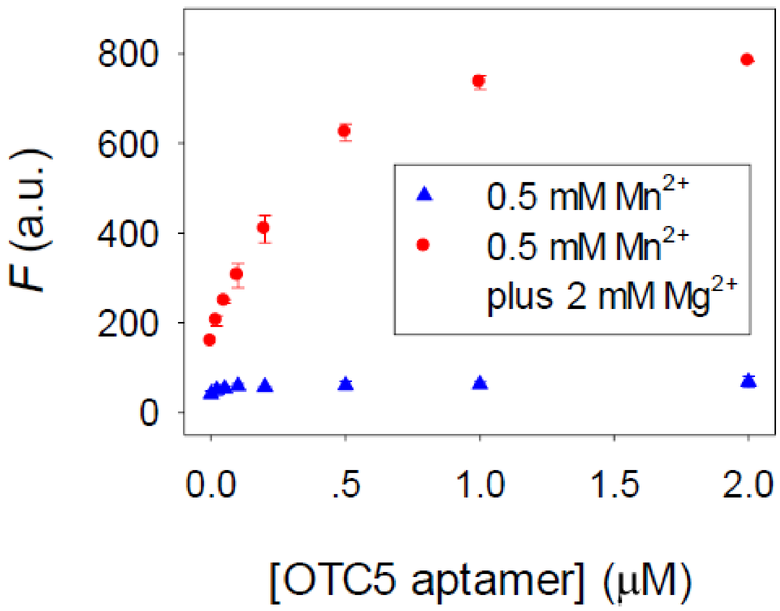

Figure S2. The fluorescence of 100 nM OTC as a function of OTC5 aptamer concentration in 0.5 mM Mn2+ alone and with 0.5 mM Mn2+ and 2 mM Mg2+ mixture. The buffer was 10 mM MES, pH 6.0 with 100 mM NaCl.
